# Refractory chronic lymphocytic leukemia–associated pruritus successfully treated with nemolizumab

**DOI:** 10.1016/j.jdcr.2026.04.056

**Published:** 2026-05-05

**Authors:** Ian A. Sellars, Yash Jani, David J. Cohen

**Affiliations:** a Mercer University School of Medicine, Macon, Georgia; b Medical College of Georgia, Augusta University, Augusta, Georgia

**Keywords:** B-cell, biological therapy, general dermatology, interleukin-31, leukemia, medical dermatology, pruritus

## Introduction

Chronic pruritus is a distressing symptom that significantly impairs quality of life and is increasingly recognized as a paraneoplastic manifestation in several malignancies. Epidemiologic studies indicate that pruritus is most strongly associated with cancers of the hematopoietic system, hepatobiliary system, and skin.^1^ Although prevalence estimates vary, pruritus has been reported in up to half of patients with B-cell chronic lymphocytic leukemia (CLL), presenting either as a diffuse, generalized itch or as part of inflammatory cutaneous lesions.^1^^,^^2^

The pathophysiology of malignancy-associated pruritus is complex and not fully understood. Many pruritogenic substances have been identified, including histamine, leukopeptidases, neuropeptides, serotonin, opioids, and cytokines such as interleukin-31 (IL-31).^3^^,^^4^

Here, we describe a 72-year-old woman with B-cell CLL who developed chronic, severe generalized pruritus refractory to first-line therapies and who ultimately experienced significant improvement with the IL-31 receptor antagonist nemolizumab. To our knowledge, use of nemolizumab for chronic lymphocytic leukemia–associated malignancy-associated pruritus has not previously been reported. This case extends the potential therapeutic scope of IL-31 receptor antagonism beyond its currently approved indications.

## Case presentation

**Fig 1:**
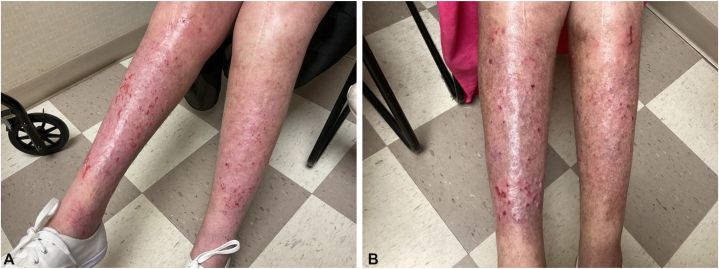
**A,** Erythematous patches with excoriated papules and erosions on the extremities consistent with secondary changes from chronic pruritus and **(B)** anterior view.

A 72-year-old female with B-cell chronic lymphocytic leukemia (CLL) diagnosed in 2007 presented with an 8-year history of severe generalized pruritus. Her treatment for CLL was initiated in 2012 and was ultimately started on ibrutinib, a Bruton tyrosine kinase inhibitor, with a good hematologic response. In October 2017, she developed persistent pruritus. A skin biopsy in December 2017 was interpreted as an infectious process and treated with antibiotics. She was subsequently managed by infectious disease and started on monthly intravenous immunoglobulin despite normal serum immunoglobulin levels. Her cutaneous lesions persisted. Serial laboratory investigations were unremarkable, with no evidence of cholestasis, renal insufficiency, thyroid dysfunction, iron deficiency, or eosinophilia to suggest alternative systemic causes of pruritus. No medications temporally associated with onset or worsening of pruritus were identified. Her CLL progressed in April 2024, prompting a change in therapy to venetoclax and rituximab. Around the same time, her pruritus intensified, and she was referred to dermatology. Examination revealed erythematous patches with excoriated papules and erosions on the trunk and extremities (Fig 1). Biopsy of a forearm lesion demonstrated features consistent with prurigo nodularis without evidence of leukemic infiltrate or primary inflammatory dermatosis, supporting secondary pruriginization from chronic systemic itch. Given her unrevealing systemic workup and clinical course, her pruritus was attributed to malignancy-associated pruritus secondary to B-cell CLL.

Multiple therapies were trialed sequentially for symptom control. She received topical corticosteroids and over-the-counter antihistamines with minimal benefit. Topical calcineurin inhibitors were also trialed without improvement. Additionally, prior systemic therapies including a serotonin-norepinephrine reuptake inhibitor and amitriptyline had been administered by an outside provider but were discontinued due to poor tolerability and lack of symptom relief. At dermatology follow-up in September 2024, her peak itch numeric rating scale (NRS) was 7/10, and her topical regimen was intensified. In November 2024, hydroxyzine and dupilumab were added according to adult atopic dermatitis guidelines, targeting Th2-mediated cytokine pathways implicated in malignancy-associated pruritus, but her pruritus worsened to an NRS of 8/10. In February 2025, gabapentin was initiated at 100 mg nightly and titrated to 300 mg nightly; however, further dose escalation was limited by tolerability, and her pruritus worsened to 10/10, significantly impairing sleep. At follow-up in April 2025, her pruritus remained refractory to dupilumab with an NRS of 8/10.

Given the severity and poor response to multiple therapies, the joint decision was made to initiate treatment with nemolizumab in July 2025. Nemolizumab was administered with a 60 mg loading dose followed by 30 mg injection after 2 weeks and then 30 mg injection every 4 weeks. At initiation, her itch NRS was 10/10. One month later, in August 2025, she reported greater than 50% improvement in symptoms, with improved sleep, decreased excoriations, and itch NRS of 3/10. Nemolizumab was well tolerated without adverse effects. At follow-up in December 2025, her symptoms were still well-controlled on nemolizumab, with a reported itch NRS of 3/10.

**Fig 2:**
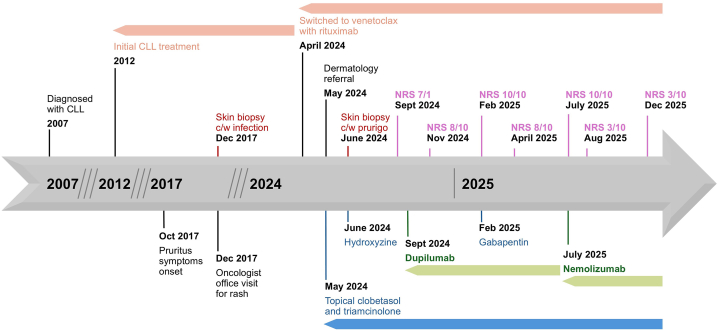
Clinical course timeline. *Orange* = oncology treatment; *purple* = itch severity (NRS); *red* = skin biopsy; *blue* = pruritus-directed treatment; *green* = biologic therapy.

A timeline of the patient’s clinical course, hematologic therapies, and itch severity is presented in Fig 2.

## Discussion

Malignancy-associated pruritus (MAP) is a debilitating symptom defined by itch lasting longer than 6 weeks that arises as a direct or paraneoplastic consequence rather than primary dermatologic disease.^1^ Its impact on sleep, mood, and daily functioning can be profound, especially in B-cell chronic lymphocytic leukemia (CLL), where MAP is recognized but likely underreported. In hematologic malignancies, paraneoplastic pruritus may precede diagnosis, correlate more closely with underlying disease biology than tumor burden, and responds incompletely to antihistamines alone.^1^ Our patient’s severe, refractory pruritus despite standard therapies highlights an important unmet therapeutic need and supports the clinical relevance of mechanism-based, targeted treatment strategies. These features highlight the need for mechanism-based therapeutic approaches.

Treatment of MAP is typically stepwise and interdisciplinary. Guidelines recommend emollients and trigger control, then H1-antihistamines, gabapentinoids, topical or short courses of systemic corticosteroids, and phototherapy; in parallel, control of the underlying malignancy is emphasized because pruritus often improves as disease activity declines.^2^ Phototherapy was not pursued in this case due to the patient’s ongoing oncology care and the additional logistical burden and time commitment associated with frequent treatment sessions. The itch-scratch cycle can even lead to secondary conditions such as prurigo nodularis as seen in this patient. Case literature has reported successes with dupilumab (IL-4Rα blockade) in CLL-associated MAP, exaggerated response to insect bites, and eosinophilic dermatosis of hematologic malignancy.^3^^,^^4^ Failures also occur, as in our patient, further emphasizing the heterogeneity of cytokine drivers in MAP. Although additional agents may be considered in chronic pruritus, management in this case favored escalation to targeted, mechanism-directed therapy given the patient’s severe symptoms and lack of response to multiple prior treatments. Neurokinin-1 receptor antagonists such as aprepitant have demonstrated efficacy in refractory malignancy-associated pruritus; however, treatment was directed toward cytokine-mediated pathways.

Mechanistically, MAP is multifactorial, with proposed mechanisms involving tumor-driven cytokine imbalance together with peripheral and central neural sensitization. Growing evidence implicates Th2-polarized cytokines, particularly interleukin-31 (IL-31), as proximate drivers of chronic itch across hematologic cancers.^5^ IL-31 and its receptor (IL-31RA) are found to be overexpressed in pruritic diseases and signal through JAK/STAT pathways in neurons and keratinocytes to amplify pruritus.^6^ Nemolizumab is a monoclonal antibody that antagonizes IL-31RA, thereby inhibiting IL-31 signaling pathways, and has demonstrated rapid, clinically meaningful itch reduction and skin improvement for atopic dermatitis and prurigo nodularis.^7^ It also contributes to maintaining skin barrier integrity, which is often disrupted during the itch–scratch cycle. The rapid and sustained response to nemolizumab suggests IL-31 signaling may have been a dominant contributor to her symptoms.

In conclusion, malignancy-associated pruritus poses a significant therapeutic challenge in patients with hematologic malignancies. This case highlights nemolizumab as a potential mechanism-based treatment option for refractory pruritus in the setting of chronic lymphocytic leukemia.

## Conflicts of interest

None disclosed.
